# Analyzing Suicide Risk From Linguistic Features in Social Media: Evaluation Study

**DOI:** 10.2196/35563

**Published:** 2022-08-30

**Authors:** Cecilia Lao, Jo Lane, Hanna Suominen

**Affiliations:** 1 School of Computing College of Engineering and Computer Science The Australian National University Canberra, ACT Australia; 2 National Centre for Epidemiology and Population Health College of Health and Medicine The Australian National University Canberra, ACT Australia; 3 Department of Computing Faculty of Technology University of Turku Turku Finland

**Keywords:** evaluation study, interdisciplinary research, linguistics, machine learning, mental health, natural language processing, social media, suicide risk

## Abstract

**Background:**

Effective suicide risk assessments and interventions are vital for suicide prevention. Although assessing such risks is best done by health care professionals, people experiencing suicidal ideation may not seek help. Hence, machine learning (ML) and computational linguistics can provide analytical tools for understanding and analyzing risks. This, therefore, facilitates suicide intervention and prevention.

**Objective:**

This study aims to explore, using statistical analyses and ML, whether computerized language analysis could be applied to assess and better understand a person’s suicide risk on social media.

**Methods:**

We used the University of Maryland Suicidality Dataset comprising text posts written by users (N=866) of mental health–related forums on Reddit. Each user was classified with a suicide risk rating (no, low, moderate, or severe) by either medical experts or crowdsourced annotators, denoting their estimated likelihood of dying by suicide. In language analysis, the Linguistic Inquiry and Word Count lexicon assessed sentiment, thinking styles, and part of speech, whereas readability was explored using the *TextStat* library. The Mann-Whitney *U* test identified differences between at-risk (low, moderate, and severe risk) and no-risk users. Meanwhile, the Kruskal-Wallis test and Spearman correlation coefficient were used for granular analysis between risk levels and to identify redundancy, respectively. In the ML experiments, gradient boost, random forest, and support vector machine models were trained using 10-fold cross validation. The area under the receiver operator curve and *F*_1_-score were the primary measures. Finally, permutation importance uncovered the features that contributed the most to each model’s decision-making.

**Results:**

Statistically significant differences (*P*<.05) were identified between the at-risk (671/866, 77.5%) and no-risk groups (195/866, 22.5%). This was true for both the crowd- and expert-annotated samples. Overall, at-risk users had higher median values for most variables (*authenticity*, *first-person pronouns*, and *negation*), with a notable exception of *clout*, which indicated that at-risk users were less likely to engage in social posturing. A high positive correlation (ρ>0.84) was present between the part of speech variables, which implied redundancy and demonstrated the utility of aggregate features. All ML models performed similarly in their area under the curve (0.66-0.68); however, the random forest and gradient boost models were noticeably better in their *F*_1_-score (0.65 and 0.62) than the support vector machine (0.52). The features that contributed the most to the ML models were *authenticity*, *clout*, and *negative emotions*.

**Conclusions:**

In summary, our statistical analyses found linguistic features associated with suicide risk, such as social posturing (eg, *authenticity* and *clout*), *first-person singular pronouns*, and *negation*. This increased our understanding of the behavioral and thought patterns of social media users and provided insights into the mechanisms behind ML models. We also demonstrated the applicative potential of ML in assisting health care professionals to assess and manage individuals experiencing suicide risk.

## Introduction

### Background

Suicide is one of the leading causes of death worldwide [1] and is an international public health problem. The World Health Organization estimates that approximately 800,000 people die because of suicide every year, and global targets to reduce suicide mortality are unlikely to be met [2].

Effective suicide risk assessment screening methods are key to reducing this preventable cause of death [3,4]. Traditional approaches to suicide risk assessment include a comprehensive clinical evaluation and the use of self-reported measures, including the Columbia Suicide Severity Rating Scale, Patient Health Questionnaire, and other measures that screen for depression and psychological distress [1,5,6]. Although these approaches provide the best practice for suicide risk assessment, not all people experiencing thoughts of suicide or suicidal ideation disclose their risk or have access to health care professionals.

In addition, people experiencing suicide risk may not seek mental health support [7,8], and for those who do, the demand for clinicians often exceeds the supply, especially in remote areas where access to health care professionals is limited [9]. Therefore, an automated risk detection tool, or a deeper understanding of the linguistic features associated with suicide risk, could allow individuals to assess their own risk of suicide. This may prompt them to seek support and, in turn, increase suicide prevention.

### Social Media and Suicide Risk Detection

Suicidal ideation has been widely documented on social media [10]. As these platforms provide individuals with an outlet to express their innermost thoughts [10], social media data offer new ways of understanding and assessing suicide risk. Hence, this creates novel possibilities for suicide assessment, intervention, and prevention [11].

Reddit, a web-based forum with >52 million daily users, offers particularly rich data. This is because of several reasons. First, it has a high character limit of 40,000 per post, which is a notable increase from other social media sites such as Twitter (280 characters), allowing users to write linguistically richer posts. Second, the website has the potential to be anonymous. Users can make *throwaway* accounts—temporary identities separate from their main accounts—to uninhibitedly discuss sensitive topics and emotions. This feature has been proven to promote open conversations and emotionally engaging feedback [12], thus making it ideal for suicide risk detection studies. Finally, Reddit’s structure is advantageous. The website is made up of subforums (subreddits) that are topic specific. This allows researchers to preselect data from mental health–related subreddits, identifying users who potentially express suicide risk.

### Machine Learning for Mental Health

In recent years, there has been increased interest in using machine learning (ML) to detect mental health conditions, including depression [13]. However, such studies often focus primarily on the performance of the classifier rather than on processes that underpin or explain its classification decisions [14].

This raises a key problem. ML models are often opaque, with *black box* models such as neural networks being largely uninterpretable [15]. This highlights a clear need for increased interpretability and understanding of the features themselves. Model-agnostic methods for understanding the feature importance include permutation importance [16] and Shapley Additive Explanations [17]. Such techniques are beneficial as they help us understand not only the outcomes but also the mechanisms behind the models themselves.

### Research Objectives

This study aimed to examine the relationship between linguistic features and indicators of users’ suicide risk on Reddit, thereby increasing interpretability. In addition to identifying statistically significant relationships, this study explored the contributions of the features to classifications by constructing ML models and permutation importance analysis.

Our main contributions are as follows: (1) we conducted nonparametric statistical analysis to identify linguistic features significantly associated with suicide risk; (2) we performed correlation analysis to identify relationships between significant features, thus identifying redundancies; (3) we built several ML models using linguistic features, highlighting the potential for future application; and (4) we measured the features that contributed the most to each model’s decision-making through permutation importance analysis.

## Methods

### Data Selection and Access

In this work, we used the existing University of Maryland Suicidality Dataset [9,18]. This comprised social media posts annotated by mental health experts and crowdsourced annotators with respect to the author’s suicide risk.

We chose this source for the following 3 main reasons.

First, it was extracted from the web-based Reddit forum. As stated earlier, Reddit has a generous character limit that allows greater linguistic complexity. Thus, it would be ideal to explore our first research question.

Second, another benefit of this data set was its high-quality annotations. A prevalent problem with social media data is the reliability of ground truth labels; it is difficult to determine whether a web user is actually at risk in real life. Annotators are often inaccurate, even when label definitions are shown [19]. The Maryland data set alleviated this issue in several ways. To begin with, the researchers preselected at-risk (low-, moderate-, and severe-risk) users by identifying people who posted on mental health–related forums (eg, SuicideWatch). Furthermore, the annotation process was completed by mental health experts and crowdsourced annotators. Consensus mechanisms (eg, multiple annotators for each user) were also used.

### Ethics Approval

The University of Maryland Suicidality Dataset [9,18] was approved for use by the Australian National University Human Research Ethics Committee (protocol number 2021/047). This was followed by obtaining proper permission to access and use it for the purposes of this study from the University of Maryland.

### Data Overview

Reddit is a web-based forum designed to help people “detach from their real-world identities” [20]. The Maryland data set comprises text posts written by 934 unique users of this website—specifically, posts published on the SuicideWatch subforum from January 1, 2008, to August 31, 2015. It includes posts from both SuicideWatch and users’ other non–mental health–related posts. In addition, users who did not post on any mental health–related forum [21] were included as a control group.

Although Reddit is intended to be anonymous, users may provide personal identifying information. Thus, this data set was further anonymized by replacing each username with a token, as well as by replacing all URLs [9,18].

### Annotation Process

The Maryland researchers annotated the data set as follows. First, posts written by a given user were temporally organized and split into annotation units. These contained up to 5 posts each. Each unit was then annotated with a suicide risk rating by either medical experts or crowdsourced contributors. Experts were given *short* instructions asking them to follow their formal training in assessing patients at risk of suicide. Meanwhile, the crowdsourced annotators were given *long* instructions that asked them to focus on risk factors such as thoughts (eg, suicide ideation and feeling like a burden), thought patterns (eg, sense of agitation), logistics (eg, talking about methods of attempting suicide), and context (eg, previous attempts and isolation from family and friends) [9,18].

Ratings were on a 4-point risk scale as follows [9]: (1) no risk (“I don’t see evidence that this person is at risk for suicide”), (2) low risk (“There may be some factors here that could suggest risk, but I don’t really think this person is at much risk of suicide”), (3) moderate risk (“I see indications that there could be a genuine risk of this person making a suicide attempt”), and (4) severe risk (“I believe this person is at high risk of attempting suicide in the near future”).

Users with <10 posts and users whose posts had <3 annotators were eliminated from the data set by the Maryland researchers. This resulted in a final sample size of 866 unique users who posted on SuicideWatch, which is described by Reddit as a peer support forum for anyone struggling with suicidal thoughts. There was also an equal number of unannotated control users (n=866). Of the 866 annotated users, 245 (28.3%) were labeled by experts, whereas 621 (71.7%) were disjointly labeled by crowdsourced contributors.

The expert annotators included multiple mental health professionals. These included a cochair of the National Suicide Prevention Lifelines Standards, Training and Practices Sub-Committee, and a clinician in the Department of Emergency Psychiatry at Boston Children’s Hospital [9,18]. To generate user-level annotations, maximum likelihood estimation was used [22,23]. Overall, the average Krippendorff interannotator agreeability α was .812.

In contrast, the crowdsourced task was completed on the web-based platform, CrowdFlower. The website’s inbuilt consensus mechanism was used to resolve disagreements among crowdsourced annotations [18]. Each user was assigned a *trust score*, which indicated their reliability. Annotations were then weighted by this trust score and aggregated into a *confidence score* for each label. The label with the highest confidence score was chosen. This resulted in a Krippendorff α of.554.

An example of a typical post is presented in (Table 1). To preserve user privacy, the post body was an aggregate of several existing posts, and the subreddit was randomly chosen.

**Table 1:** Example of a typical Reddit post from the data set and the suicide rating.

Features	Value
Post ID	1a2b3c
User ID	45678
Time stamp (Unix epoch)	1.4E+09
Subreddit	r/self-harm
Post body	“I’ve been feeling depressed for a while. I don’t know how to deal with it anymore...”
Label	Severe risk

### Data Preprocessing and Linguistic Feature Engineering

In our research, we randomly split the data into an 80:20 training-test ratio following the Pareto principle [24]. This was achieved by randomly selecting 80% of the user IDs from both the crowd- and expert-annotated data sets. All posts associated with the users were then retrieved. In addition, unannotated control users and posts without any text were discarded. For the ML models, expert- and crowd-annotated users were combined into singular training and test sets to maximize the available data. Meanwhile, the statistical analysis was performed on the expert and crowd data sets separately to compare the distributions of the different groups.

**Figure 1:**
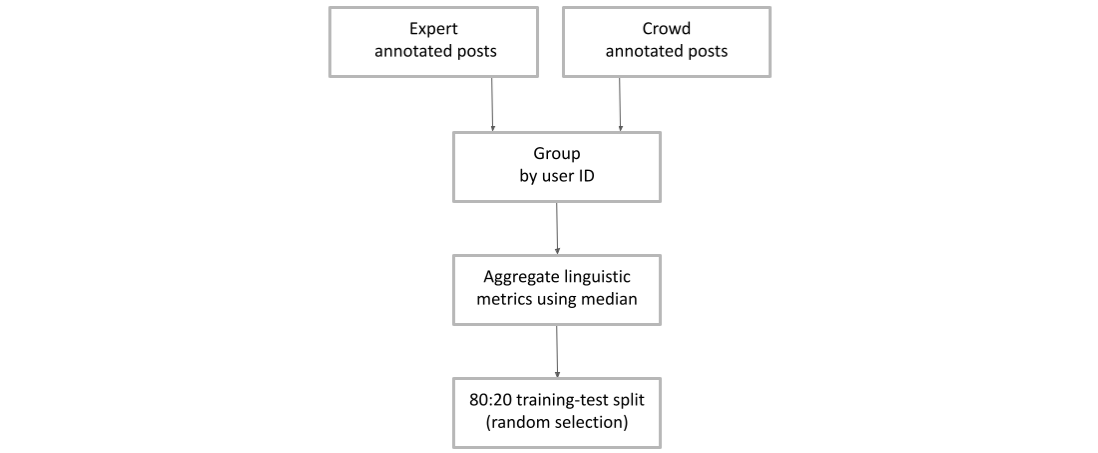
Flowchart detailing the data preprocessing stages.

Linguistic features for users were aggregated by taking the average of all the posts (Figure 1). The median rating was used instead of the mean rating to reduce the influence of outliers. Overall, we chose to group according to users to reflect the annotation process, as ratings were attached to a user rather than an individual post.

Linguistic Inquiry and Word Count (LIWC) 2015 and the *TextStat* Python library were used to extract linguistic features from posts. All the LIWC and *TextStat* features are listed in Multimedia Appendix 1 and Multimedia Appendix 2, respectively.

LIWC is a lexicon [25] that groups words into psychologically meaningful categories. Aside from aggregate features such as *authenticity*, the scores for most features were the percentage of total words in a text that belonged to a specific category. Prior studies have demonstrated the capacity of LIWC to detect emotionality [26,27], thinking styles [28], and individual differences [29,30]. Moreover, it has been used to detect self-reported symptoms of depression and other mental health conditions [31,32]. In this study, all the categories were used to ensure comprehensive coverage.

In juxtaposition, *TextStat* is a computerized analysis tool that measures linguistic complexity. This package was selected because it contains both simple features such as word count and widely used linguistic readability metrics such as the Gunning Fog Index, Simple Measure of Gobbledygook, and Flesch-Kincaid scores.

### Statistical and Correlation Analyses

Statistical and correlation analyses were designed and reported in consultation with the Australian National University Statistical Consultation Unit.

Statistical errors because of the assumption of normality are common in quantitative studies [33]. To mitigate this, we used the Shapiro-Wilk test [34] from *scipy.stats*. The analysis revealed that none of the features were normally distributed. Hence, nonparametric tests were used to compare the distributions of different risk groups.

First, a 2-sided Mann-Whitney *U* test was used to compare at-risk and no-risk users. This test was chosen because of its nonparametric nature and previous applications in medical studies [35]. To form this binary grouping, users who received either a severe-, moderate-, or low-risk rating were considered at risk. Meanwhile, users who received a no-risk rating formed their own group.

To supplement these results, we used the Kruskal-Wallis test. This compared the distribution of features within different risk levels. This analysis allowed us to determine whether the severe-, moderate-, and low-risk groups behaved differently.

For both tests, we used an α value of *P*<.05. To correct for multiple comparisons, we applied the Benjamini-Hochberg procedure [36] to the *P* values. We calculated 95% CIs to estimate the difference between medians using the Mann-Whitney *U* test. In addition, although post hoc methods (eg, Dunn test) for the Kruskal-Wallis test can be calculated to determine which specific medians are different, these were not computed in this work, as this was largely observed through the use of comparative box plots. Python (*scipy.stats* and *scipy.statsmodels*) and R (*wilcox* and *kruskal*) libraries were used for the implementation.

To reach a consensus between the expert- and crowd-annotated data sets, the features needed to have *P* values of <.05 and the same directionality to be labeled as *significant* in the Mann-Whitney *U* and Kruskal-Wallis tests. In addition, features with 95% CIs that included 0 were eliminated. This was because of 2 main reasons.

First, crowdsourced annotators were less reliable than experts. As they had less training, they had a lower macro *F*_1_-score, with a tendency to misclassify lower-risk users as having higher risk [9].

Second, the distribution of features could be different because of random variation. Although this does not necessarily mean that features that are only significant in one data set are not significant overall, it does suggest that the distributions are noticeably different. Therefore, it would be inappropriate to compare them.

A correlation analysis was also used to identify redundancies. This was because, in a practical context, having too many features limits interpretability and increases the computational complexity. For instance, if there are thousands of features, even if we know the weighted contribution of each feature, it is still extremely difficult to fully understand ML models and their classifications [37]. Hence, we identified relationships between significant features to determine potential proxies on both the expert- and crowd-annotated data sets, with *P* values corrected using the Benjamini-Hochberg procedure. We selected the Spearman correlation coefficient because of its nonparametric nature [38] and established use in medical research [39].

### ML Models, Their Performance Evaluation, and Feature Importance Analysis

To determine whether the features would prove useful for risk assessment, we constructed several preliminary ML models that classified whether a user was at risk or had no risk. For this project, we used random forest (RF) [40], gradient boost (GB) [41], and support vector machines (SVMs) [42]. These techniques were selected because of their application in mental health research [43]. All LIWC and *TextStat* features were used to train the models.

An SVM is a supervised ML algorithm. It classifies data by representing each data point as a vector and fitting a hyperplane that separates the different classes [42]. In a 2D context, this is equivalent to fitting a dividing line through the data. Intuitively, an optimal hyperplane in such a fitting should be approximately at the center of the 2 classes. For SVMs, this is determined by calculating the distance between the hyperplane and the closest data points from each class. The hyperplane that maximizes this distance, or the maximum-margin hyperplane, is selected [42]. As not all data are linearly separable [44], SVMs use a kernel function in classification problems, a mathematical operation that performs the equivalent of mapping a lower-dimensional space to a higher dimension [45]. Ideally, this higher-dimensional projection should help make the data separable and, therefore, classifiable.

Decision trees are nonparametric and supervised learning methods. They work by splitting the root node, which represents the entire data set, into branch-like segments based on the values of their features. This continues until all the data are matched to a leaf node, which represents a class label [46]. Splitting is determined by the *purity* of the split, which is measured by metrics such as the information gain, gini index, and gain ratio [47].

Fundamentally, the algorithm tries to split the data so that the data points in each branch belong to the same class. However, a problem with decision trees is that they are prone to overfitting [48]. Hence, a common way of addressing this problem is through ensemble methods that combine multiple smaller classifiers into a single classifier [49].

The RF method is a prime example of an ensemble method. It works by drawing *k* random subsamples of the data and fitting decision trees to each subsample. When presented with a new data point, each of the *k* decision trees casts a vote for the class label. The final label is determined by the results of the majority vote [40].

GB is another decision tree–based ensemble method. However, in contrast to RF, GB functions in an additive manner [50]. Fundamentally, this implies that each of the *k* decision trees is iteratively trained. The first decision tree is fitted to the training data, and the error is calculated. Following this, data points that were incorrectly classified will be given a higher weight, so that the following model can address the deficiencies of the previous model [41]. After all the weak learners have been trained, the final class label is determined by a weighted majority vote, with votes from more successful learners being more important.

As noted above, an 80:20 training-test split was used. The class distribution was as follows. In the training set, there were 63% (546/866) at-risk and 16.4% (142/866) no-risk users. A similar distribution was observed in the test set, with 14.4% (125/866) at-risk and 6.1% (53/866) no-risk users. To find the optimal hyperparameters and reduce overfitting [51], we used 10-fold cross validation [52] on the training set. The area under the receiving operator curve (AUC) was used as the primary scoring metric for validation because of increased discrimination and consistency [53,54]. To evaluate the performance on the test set, a more diverse range of metrics, including the AUC and accuracy, as well as the precision, recall, and *F*_1_-score, were used to balance the trade-off between sensitivity and specificity [55]. Finally, confusion matrices [56] provided visualizations of true and false positives, as well as negatives on the test set.

Although univariate statistical tests can uncover relationships between linguistic variables and suicide risk, they might not indicate the importance of features in a given ML model. Hence, to better understand our models’ decision-making process, we analyzed the permutation importance of each feature [16]. This examined the decrease in an existing model’s score over a given number of iterations when the values of a single feature were randomly reordered. We implemented this using the Python *sklearn* library.

Features were considered important for a given model if a large decrease was observed and vice versa. For the purposes of this research, we calculated the permutation importance over 100 iterations on a holdout test set and used AUC, precision, and recall as the scoring mechanisms. Only variables with mean permutation importance values >1 SD away from 0 were considered significant.

## Results

### Mann-Whitney U Test

At-risk (low-, moderate-, and severe-risk) users had, on average, a greater use of *authenticity*, *first-person singular pronouns*, and *negation* (Multimedia Appendices 3 and 4). They also had lower *clout* (Tables 2 and 3). This suggests that, overall, they were more authentic in their expression and engaged less in social posturing (*authenticity* and *clout*). For brevity, all tables show only statistically significant values (*P<*.05) after applying the Benjamini-Hochberg correction, with the mean and CIs rounded to 4 significant figures.

**Figure 2:**
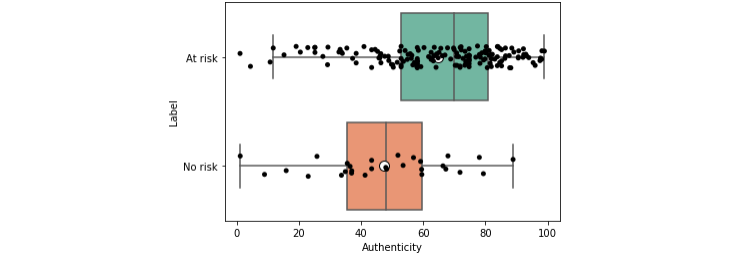
Box plot for authenticity for at-risk and no-risk users (expert).

**Figure 3:**
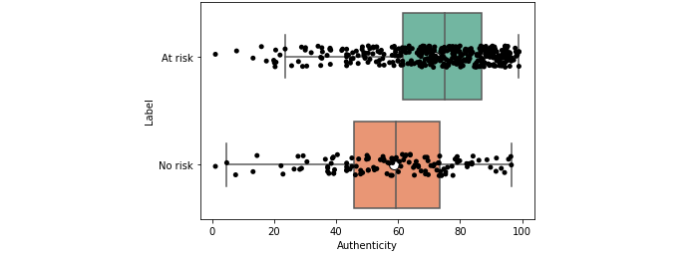
Box plot for authenticity for at-risk and no-risk users (crowd).

**Figure 4:**
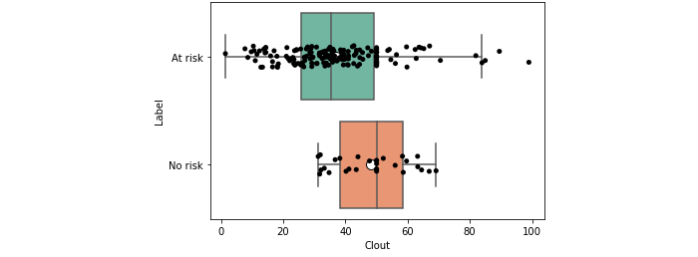
Box plot for clout for at-risk and no-risk users (expert).

**Figure 5:**
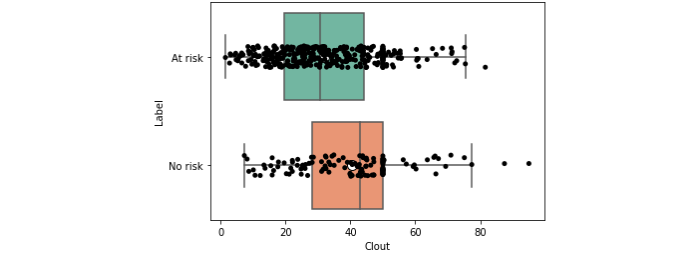
Box plot for clout for at-risk and no-risk users (crowd).

This observation was also reflected visually. The box plots, which show that the overall distributions, in addition to the central measures such as the mean and median, were skewered further left for the at-risk users (Figures 2 and 3). Again, the inverse was observed for clout (Figures 4 and 5).

**Table 2:** Mann-Whitney U test results for expert-annotated users.

Feature	Examples	At-risk, mean (SD)	No-risk, mean (SD)	*P* value	95% CIs for differences between medians
Clout	N/A^a^	36.81 (16.62)	48.21 (11.48)	.005	−17.83 to −6.590
Authenticity	N/A	64.82 (21.31)	47.35 (20.86)	.005	10.04 to 27.09
First-person singular pronouns	I, my, and mine	7.105 (2.979)	5.419 (2.194)	.04	0.6900 to 2.840
Negation	Not, no, and never	1.391 (0.8524)	0.7924 (0.6987)	.01	0.2250 to 0.9650

^a^N/A: not applicable.

**Table 3:** Mann-Whitney U test results for crowd-annotated users.

Feature	Examples	At-risk, mean (SD)	No-risk, mean (SD)	*P* value	95% CIs for differences between medians
Clout	N/A^a^	32.00 (15.71)	40.48 (16.42)	<.001	−12.29 to −5.315
Authenticity	N/A	71.66 (19.57)	58.73 (20.18)	<.001	9.985 to 17.75
First-person singular pronouns	I, my, and mine	8.346 (2.902)	6.738 (2.579)	<.001	1.120 to 2.195
Negation	Not, no, and never	1.717 (1.072)	1.284 (1.031)	.001	0.1500 to 0.6000

^a^N/A: not applicable.

### Kruskal-Wallis Test

When comparing the severe-, moderate-, low-, and no-risk groups (Multimedia Appendices 5 and 6), no LIWC or *TextStat* features were significant (*P*<.05) for both the expert- and crowd-annotated data sets after correction. This indicates that although certain linguistic variables are associated with at-risk versus no-risk groups, there are no significant differences within the at-risk groups themselves.

### Correlation Analysis

Using the Spearman correlation coefficient (Multimedia Appendices 7-10), we found the following.

For LIWC, various parts of speech (eg, *function words* and *pronouns*; *ρ*>0.74 and 0.84 [unless otherwise specified, the first *ρ* is the correlation coefficient on the crowd-annotated data set, whereas the second is for the expert-annotated data set; all figures have been rounded to 2 significant figures]) were highly correlated with each other, indicating redundancy.

In addition, many parts of speech also had moderate correlations with variables measuring post length, such as syllable count (eg, *comparatives*; *ρ*>0.50 and 0.63) and word count (eg, *focus on the future*; *ρ*>0.59 and 0.67). This suggests that they could be proxies of length. Intuitively, this association was understandable, as the longer the post, the more parts of speech it will have.

Some features such as *clout* and *authenticity* appeared to be aggregate features, combining other variables, as shown through moderate positive correlations with other variables (eg, *authenticity* and *function words*; *ρ*>0.47 and 0.59). This was in line with the LIWC manual [57] and suggested that overall, aggregate features could be an efficient way of condensing information. Visual and statistical evidence provided additional support for this conclusion. When examining the box plots (Figures 2-5), we found that the aggregate measures were generally more discernible than individual categories. Furthermore, the *P* values tended to be smaller (*P*<.01), and the distance between medians also tended to be greater (Tables 2 and 3).

### Model Performance

Overall, all models showed great promise in identifying suicide risk and achieved a similar performance (Table 3; Multimedia Appendices 11 and 12). Most errors lay in a tendency to overassign to the at-risk category. As type II errors are preferable to their type I counterparts in the medical domain, this demonstrated that LIWC and *TextStat* features are effective for building ML risk assessment models.

The performances of all models were largely comparable, with the AUCs for the GB, RF, and SVM models being 0.67, 0.66, and 0.68, respectively. Furthermore, when looking at performance evaluation outcomes (Table 4; Multimedia Appendices 11 and 12), all models were better at classifying at-risk users, with the RF model having the highest performance (*F*_1_-score of 0.83) for this class. This was most likely because of the imbalanced nature of the data, with more users being at risk than not because of the selection process. It should be noted that the SVM, in particular, performed worse on the no-risk class, as indicated by its noticeably lower *F*_1_-score (0.52). This implies that it is less useful in practice.

**Table 4:** Summary of classification results of various machine learning models^a^.

Models	AUC^b^	Accuracy	Precision	Recall	*F*_1_-score
Gradient boost	0.67	0.62	0.61	0.67	0.62
Random forest	0.66	0.75	0.65	0.66	0.65
Support vector machine	0.68	0.53	0.64	0.68	0.52

^a^The precision, recall, and *F*_1_-scores are the macroaverage of the different classes.

^b^AUC: area under the receiving operator curve.

### Permutation Importance

A noticeable overlap was present in the features that had higher permutation importance for the GB and RF models (Table 5), with *authenticity*, *negative emotion*, and *clout* contributing to higher precision and AUC (*authenticity* and *negative emotion* only) for both models. Meanwhile, the SVM yielded different results, having no common significant features with other models. However, as noted earlier, the SVM model had a notably lower performance (*F*_1_-score) than the other 2 models. The permutation importance only measures the importance of a feature for a given model. Hence, if a model did not perform well, its permutation importance analysis results were not necessarily reliable. Thus, rather than showing that the aforementioned features were not important, this disparity could be an indicator of model quality.

**Table 5:** Permutation importance results for AUC^a^, precision, and recall.

Features		Gradient boost, mean (SD)	Random forest, mean (SD)	Support vector machine, mean (SD)
**AUC**				
	Authenticity	0.071 (0.041)	0.041 (0.027)	N/A^b^
	Negative emotion	0.034 (0.024)	0.017 (0.01)	N/A
	Clout	0.02 (0.016)	N/A	N/A
	Whitespace	N/A	N/A	0.01 (0.005)
**Precision**				
	Authenticity	0.057 (0.026)	0.030 (0.013)	N/A
	Clout	0.018 (0.013)	0.035 (0.012)	N/A
	Negative emotion	0.016 (0.014)	0.020 (0.011)	N/A
	First-person singular pronouns	N/A	0.015 (0.008)	N/A
	Quantitative processes	N/A	N/A	0.014 (0.010)
	Informality	N/A	N/A	0.011 (0.008)
**Recall**				
	Negative emotion	N/A	0.022 (0.015)	N/A
	Positive emotion	N/A	0.021 (0.011)	N/A
	Question mark	N/A	0.016 (0.007)	N/A
	Affect	N/A	0.013 (0.008)	N/A
	Function words	N/A	0.013 (0.008)	N/A
	Colon	N/A	N/A	0.011 (0.004)
	Ingest	N/A	N/A	0.01 (0.005)

^a^AUC: area under the receiving operator curve.

^b^N/A: not applicable.

## Discussion

### Principal Findings and Prior Work

A key finding was that linguistic features were significantly (*P*<.05, 95% CIs) associated with suicide risk on social media. This was achieved using nonparametric statistical analysis. Significant variables included social (*authenticity* and *clout*) and grammatical (*first-person singular pronouns* and *negation*) features (Tables 2 and 3). This confirmed prior studies linking suicide risk and depression to the increased use of first-person pronouns [31,58,59].

In addition to complementing prior work [31,58,59], our contribution provided novelty by examining the directionality and distribution of features at a finer granularity. We found that at-risk users tended to be more authentic and less concerned about social posturing (Tables 2 and 3). Overall, at-risk users had a larger median value than no-risk users for most features. However, there was no real difference between the distributions of the significant variables for low-, moderate-, and severe-risk users.

Another notable finding was the identification of redundant features such as various parts of speech. Although numerous studies have examined the relationship between linguistic features and adverse mental health [58,59], few statistically examined the correlation between the significant features themselves. Moreover, although there are mathematical methods [17,60] for determining feature importance, these techniques are not widely used in the health sciences. Hence, established methods such as the Spearman correlation coefficient may be easier for clinicians to interpret.

Through correlation analysis, we found moderate positive relationships between readability metrics (eg, *Gunning Fog Index*; *ρ*>0.77 and 0.76), parts of speech (eg, *comparatives*; *ρ*>0.50 and 0.63), and post length (eg*, syllable*, *word*, and *sentence count*). This indicates that the underlying feature, length, could potentially be used in favor of its proxies. Moreover, using aggregate variables such as *clout* and *authenticity* may further increase computational efficiency. Not only do they combine more detailed categories, but they may also be better at discerning risk levels because of the increased differences between medians.

Another contribution was the demonstration that linguistic features alone could be used to create effective ML models (GB, RF, and SVMs). After hyperparameter tuning, the models achieved commendable AUCs ranging from 0.66 to 0.68 and *F*_1_-scores ranging from 0.52 to 0.65. This received at par, if not better, performance than other lexical feature–based models whose AUC and *F*_1_-scores ranged from 0.51 to 0.75 [61] and from 0.20 to 0.32 [62], respectively. In addition, all models had a markedly better *F*_1_-score for the at-risk group (Multimedia Appendices 11 and 12). As failing to identify a person with high suicide risk could lead to loss of life, a more conservative model is advantageous for suicide prevention.

Finally, we used permutation importance to identify the features that contributed the most to each model’s decision-making. Through this analysis, we found that *authenticity* and *negative emotion* contributed to higher AUC and precision scores for both the GB and RF models, whereas *clout* contributed to a higher precision for the models. This indicated that such features could potentially be important indicators of suicide risk.

### Reproducibility

As we are not the data set owners, we will not be able to provide it upon request. Thus, all applications for data access should be directed at the University of Maryland, following their formal request protocol.

By nature, ML for mental health is a sensitive research area. Hence, the source code for our experiments and the parameters of the classifiers will be made available upon reasonable request, with a justification for the intended use. All code distribution will be under the Massachusetts Institute of Technology license.

### Limitations

Our study had 4 primary limitations. First, the observational nature of the study should be noted. Owing to privacy concerns, the University of Maryland Suicidality Dataset does not have ground truth labels, and we were unable to confirm whether users labeled as at risk were in fact experiencing suicidal ideation. In addition, it should be acknowledged that a person experiencing suicidal ideation may not be at risk of suicide, and people on the SuicideWatch forum may be affected by suicide through a family member or friend and not be experiencing suicide risk themselves. However, these confounds are likely to have been mitigated by expert annotation and consensus mechanisms.

Second, another limitation was the granularity of the annotations. Annotations were attached to each user and not to each post. Hence, we did not know which posts were more important and used aggregated features to train the models. Therefore, the performance could have potentially been further improved with finer-grained annotations.

Our third main limitation was the use of only linguistic features to train the models. As demonstrated by prior work, behavioral and relational analyses may further improve automated screening for suicide risk [35,63]. However, having a production-ready model was not the aim of this study. Instead, we aimed to determine whether simpler interpretable models could be used to screen for suicide risk. This was done to ensure that the models were accessible to health care professionals. Hence, *black box* ML methods such as deep learning and nonlexical features were not considered.

Our final limitation was the use of permutation importance to indicate the feature importance. As previously stated, permutation importance indicates only the importance of a feature for a particular model. Hence, it is arguably limited by the effectiveness of the models.

### Future Work

This study focused on highlighting the usefulness of linguistic features in constructing ML models. Hence, only lexical features were used. However, prior studies [35,63] indicate that features based on behavioral data and metadata can be used to enhance performance. Therefore, before deploying our model for production, the inclusion of a more varied range of features could be investigated. It would also be interesting to explore deep learning as this would help us evaluate whether latent variables could further increase performance.

The usefulness of our findings in practice and how they relate to suicide assessment, intervention, and prevention could also be examined. This can be done in two ways: (1) exploring the use of ML-based models to support risk assessment on social media sites themselves and (2) investigating the integration of our work into clinical practice.

**Figure 6:**
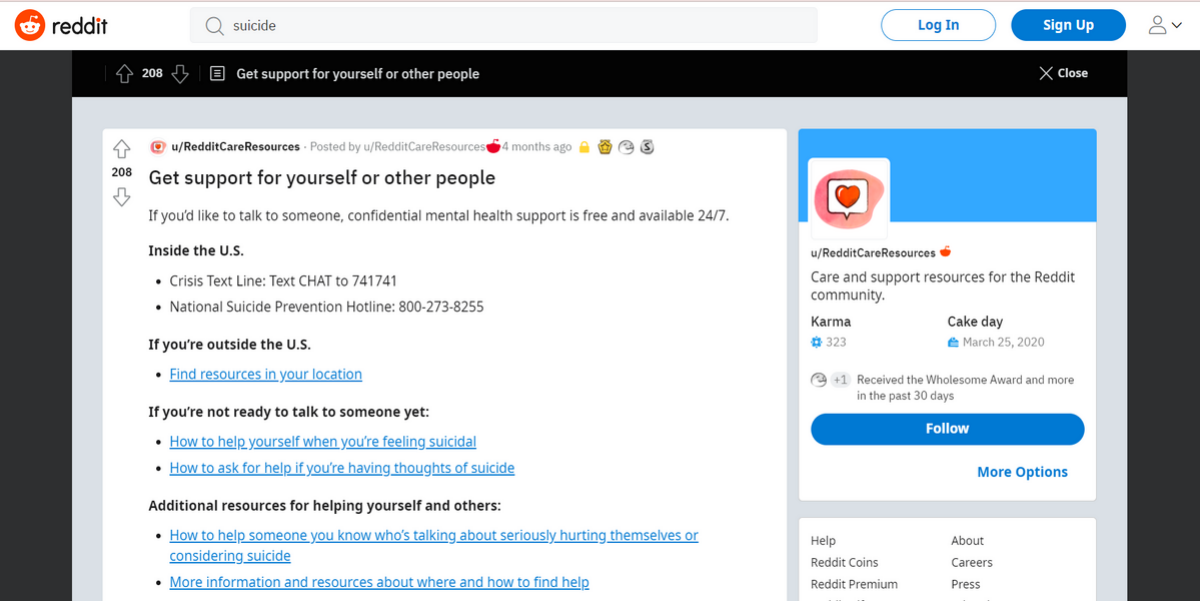
Screenshot of Reddit Care Resources.

With regard to existing interventions on social media, in March 2020, Reddit developed *Reddit Care Resources*—an initiative aimed at providing mental health resources to users at risk of suicide or self-harm [64-66]. This method operates in 2 ways. First, if a user searches for certain keywords (eg, “suicide” and “kill myself”), the first result displayed is a post indicating where to find mental health support (Figure 6). Second, users can confidentially report other users who they believe are at risk of suicide or self-harm, which then connects them to trained crisis counselors [64,65].

**Figure 7:**
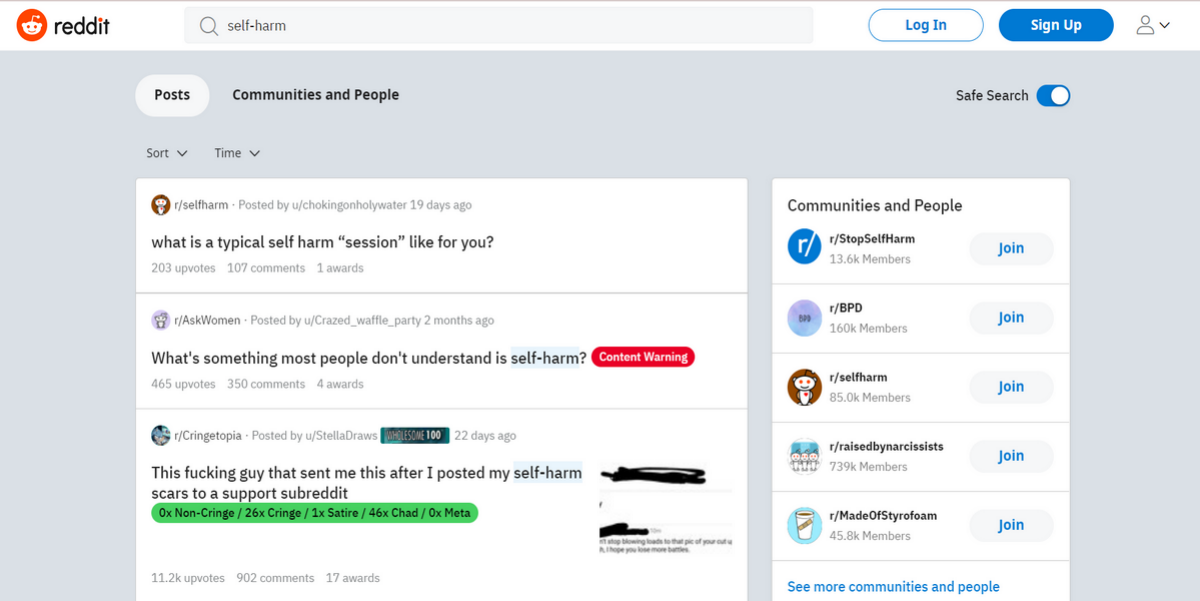
Screenshot of search results for “self-harm”.

Although these changes mark an increasing awareness of mental health and suicide risk, these measures could still be improved. For instance, the list of keywords that triggers Reddit Care Resources is limited, with searches for “depression,” “self-harm,” or “anorexia” not prompting this intervention (Figure 7).

ML models, such as those used in this study, could help alleviate this problem. For example, Reddit could run such models on searches and posts, prompting Reddit Care Resources to pop up if a certain risk threshold is met. This would eliminate the need to constantly expand the mental health–related keyword list, as internet slang and neologisms (eg, “proana” for “pro-anorexia”) can make it difficult to record every word related to mental health.

Examining how our work can be integrated into clinical practice would also be meaningful. Social media can offer an outlet for people to express opinions and thoughts that they may find difficult to express face to face [12]. Hence, analysis of such posts by a health care professional may allow for a deeper understanding of their clients if informed consent is granted. However, a problem is that directly reading such posts may result in an unintentional breach of confidentiality [67]. For instance, if a client shares web-based posts with a health care professional that includes self-harm or abuse, they may be required to report this as part of their duty of care and mandatory reporting obligations [67].

Using a combination of ML and linguistic features (eg, LIWC and *TextStat*), as demonstrated in this work, could help address this problem. Being very time poor, health care professionals do not have time to read through social media posts. Instead, with consent, automated methods could provide a report that summarizes suicide risk and other clinically relevant information, including the affective (eg, *emotional tone*), cognitive (eg, *discrepancy* and *certainty*), and social aspects (eg, *clout* and *authenticity*) of posts. This preserves client privacy while using ML to extract important clinical information that can potentially enhance client engagement and care. Furthermore, coproduction approaches with mental health experts and people with lived experience of suicide risk would help identify user and system requirements. This, in turn, would facilitate the development of future software apps (eg, desktop and mobile).

A final future development would be to diversify the annotated data sets. The University of Maryland Suicidality Dataset was unique because of its expert annotation and heightened levels of reliability; however, it has some limitations. For example, the demographics of Reddit tend to skew toward young and male [68-70], which is not representative of the world’s population. Hence, gathering a wider and more varied data set would increase the generalizability of our work. Moreover, it may be helpful to further increase the granularity of annotations. There are 2 main reasons for this. First, it would help us understand which text posts contributed the most to an annotator’s decision. Second, it would allow us to examine the fluctuation of risk within an individual, as a person identified as at risk may no longer be at risk at another point in time. These additions would likely allow us to achieve more informed results.

### Conclusions

In this study, we demonstrated the potency of linguistic features in supporting suicide risk assessment through social media posts. Through statistical and permutation analyses, we were able to determine features significantly related to suicide risk, features that contributed the most to risk classifications, and redundancy through feature relationships. Finally, the commendable performances of the SVM, GB, and RF models highlight the utility of lexical features alone. This suggests that with future development, these models could be highly useful tools to help enhance clinical care.

## Appendix

Multimedia Appendix 1 Full glossary of Linguistic Inquiry and Word Count features.

Multimedia Appendix 2 Full glossary of TextStat features.

Multimedia Appendix 3 Full list of corrected *P* values for the Mann-Whitney U test (expert).

Multimedia Appendix 4 Full list of corrected *P* values for Mann-Whitney U test (crowd).

Multimedia Appendix 5 Full list of corrected *P* values for the Kruskal-Wallis test (expert).

Multimedia Appendix 6 Full list of corrected *P* values for the Kruskal-Wallis test (crowd).

Multimedia Appendix 7 Spearman correlation coefficients for Linguistic Inquiry and Word Count and TextStat features (expert).

Multimedia Appendix 8 Spearman correlation coefficient–corrected *P* values for Linguistic Inquiry and Word Count and TextStat features (expert).

Multimedia Appendix 9 Spearman correlation coefficients for Linguistic Inquiry and Word Count and TextStat features (crowd).

Multimedia Appendix 10 Spearman correlation coefficients–corrected *P* values for Linguistic Inquiry and Word Count and TextStat features (crowd).

Multimedia Appendix 11 Classification matrix for the at-risk class for machine learning models.

Multimedia Appendix 12 Classification matrix for the no-risk class for machine learning models.

## Abbreviations

AUC: area under the receiving operator curve
GB: gradient boost
LIWC: Linguistic Inquiry and Word Count
ML: machine learning
RF: random forest
SVM: support vector machine
