# Supplementary material for: Analyzing Suicide Risk From Linguistic Features in Social Media: Evaluation Study
Source: JMIR Form Res. 2022 Aug 30;6(8):e35563. doi: 10.2196/35563 (PMC9472054; doi:10.2196/35563)
Supplement: Multimedia Appendix 1 [file formative_v6i8e35563_app1.docx]

| **Category** | **Examples** | **Words in category** | **Internal Consistency**  **(Uncorrected α)** | **Internal Consistency**  **(Corrected α)** |
| --- | --- | --- | --- | --- |
| Word count | ­ | ­ | ­ | ­ |
| **Summary Language Variables** |  |  |  |  |
| Analytical thinking | ­ | ­ | ­ | ­ |
| Clout | ­ | ­ | ­ | ­ |
| Authentic | ­ | ­ | ­ | ­ |
| Emotional tone | ­ | ­ | ­ | ­ |
| Words/sentence | ­ | ­ | ­ | ­ |
| Words > 6 letters | ­ | ­ | ­ | ­ |
| Dictionary words | ­ | ­ | ­ | ­ |
| **Linguistic Dimensions** |  |  |  |  |
| Total function words | it, to, no, very | 491 | .05 | .24 |
| Total pronouns | I, them, itself | 153 | .25 | .67 |
| Personal pronouns | I, them, her | 93 | .20 | .61 |
| 1st pers singular | I, me, mine | 24 | .41 | .81 |
| 1st pers plural | we, us, our | 12 | .43 | .82 |
| 2nd person | you, your, thou | 30 | .28 | .70 |
| 3rd pers singular | she, her, him | 17 | .49 | .85 |
| 3rd pers plural | they, their, they’d | 11 | .37 | .78 |
| Impersonal pronouns | it, it’s, those | 59 | .28 | .71 |
| Articles | a, an, the | 3 | .05 | .23 |
| Prepositions | to, with, above | 74 | .04 | .18 |
| Auxiliary verbs | am, will, have | 141 | .16 | .54 |
| Common Adverbs | very, really | 140 | .43 | .82 |
| Conjunctions | and, but, whereas | 43 | .14 | .50 |
| Negations | no, not, never | 62 | .29 | .71 |
| **Other Grammar** |  |  |  |  |
| Common verbs | eat, come, carry | 1000 | .05 | .23 |
| Common adjectives | free, happy, long | 764 | .04 | .19 |
| Comparisons | greater, best, after | 317 | .08 | .35 |
| Interrogatives | how, when, what | 48 | .18 | .57 |
| Numbers | second, thousand | 36 | .45 | .83 |
| Quantifiers | few, many, much | 77 | .23 | .64 |
| **Psychological Processes** |  |  |  |  |
| Affective processes | happy, cried | 1393 | .18 | .57 |
| Positive emotion | love, nice, sweet | 620 | .23 | .64 |
| Negative emotion | hurt, ugly, nasty | 744 | .17 | .55 |
| Anxiety | worried, fearful | 116 | .31 | .73 |
| Anger | hate, kill, annoyed | 230 | .16 | .53 |
| Sadness | crying, grief, sad | 136 | .28 | .70 |
| Social processes | mate, talk, they | 756 | .51 | .86 |
| Family | daughter, dad, aunt | 118 | .55 | .88 |

| **Category** | **Examples** | **Words in category** | **Internal Consistency**  **(Uncorrected α)** | **Internal Consistency**  **(Corrected α)** |
| --- | --- | --- | --- | --- |
| Friends | buddy, neighbor | 95 | .20 | .60 |
| Female references | girl, her, mom | 124 | .53 | .87 |
| Male references | boy, his, dad | 116 | .52 | .87 |
| Cognitive processes | cause, know, ought | 797 | .65 | .92 |
| Insight | think, know | 259 | .47 | .84 |
| Causation | because, effect | 135 | .26 | .67 |
| Discrepancy | should, would | 83 | .34 | .76 |
| Tentative | maybe, perhaps | 178 | .44 | .83 |
| Certainty | always, never | 113 | .31 | .73 |
| Differentiation | hasn’t, but, else | 81 | .38 | .78 |
| Perceptual processes | look, heard, feeling | 436 | .17 | .55 |
| See | view, saw, seen | 126 | .46 | .84 |
| Hear | listen, hearing | 93 | .27 | .69 |
| Feel | feels, touch | 128 | .24 | .65 |
| Biological processes | eat, blood, pain | 748 | .29 | .71 |
| Body | cheek, hands, spit | 215 | .52 | .87 |
| Health | clinic, flu, pill | 294 | .09 | .37 |
| Sexual | horny, love, incest | 131 | .37 | .78 |
| Ingestion | dish, eat, pizza | 184 | .67 | .92 |
| Drives |  | 1103 | .39 | .80 |
| Affiliation | ally, friend, social | 248 | .40 | .80 |
| Achievement | win, success, better | 213 | .41 | .81 |
| Power | superior, bully | 518 | .35 | .76 |
| Reward | take, prize, benefit | 120 | .27 | .69 |
| Risk | danger, doubt | 103 | .26 | .68 |
| Time orientations |  |  |  |  |
| Past focus | ago, did, talked | 341 | .23 | .64 |
| Present focus | today, is, now | 424 | .24 | .66 |
| Future focus | may, will, soon | 97 | .26 | .68 |
| Relativity | area, bend, exit | 974 | .50 | .86 |
| Motion | arrive, car, go | 325 | .36 | .77 |
| Space | down, in, thin | 360 | .45 | .83 |
| Time | end, until, season | 310 | .39 | .79 |
| Personal concerns |  |  |  |  |
| Work | job, majors, xerox | 444 | .69 | .93 |
| Leisure | cook, chat, movie | 296 | .50 | .86 |
| Home | kitchen, landlord | 100 | .46 | .83 |
| Money | audit, cash, owe | 226 | .60 | .90 |
| Religion | altar, church | 174 | .64 | .91 |
| Death | bury, coffin, kill | 74 | .39 | .79 |
| Informal language |  | 380 | .46 | .84 |
| Swear words | fuck, damn, shit | 131 | .45 | .83 |
| Netspeak | btw, lol, thx | 209 | .42 | .82 |
| Assent | agree, OK, yes | 36 | .10 | .39 |
| Nonfluencies | er, hm, umm | 19 | .27 | .69 |
| Fillers | Imean, youknow | 14 | .06 | .27 |
