# Supplementary material for: Analyzing Suicide Risk From Linguistic Features in Social Media: Evaluation Study
Source: JMIR Form Res. 2022 Aug 30;6(8):e35563. doi: 10.2196/35563 (PMC9472054; doi:10.2196/35563)
Supplement: Multimedia Appendix 2 [file formative_v6i8e35563_app2.docx]

| Feature | Formula | Interpretation |
| --- | --- | --- |
| Flesch Reading Ease Formula | $206.835-1.015(\frac{total words}{total sentences})-84.6(\frac{total syllables}{total words})$ | A score of 100 means a text is extremely easy to read, while a score 0 means the inverse. |
| Flesch Kincaid Grade Level | $0.39\frac{total words}{total sentences}+11.8(\frac{total syllables}{total words})-15.59$ | Represents the grade level needed to read the text (e.g. 9.3 = a ninth grader can read it). |
| The Fog Scale | $0.4[\frac{total words}{total sentences}+100(\frac{complex words}{total words})]$ | Represents the grade level needed to read the text (e.g. 9.3 = a ninth grader can read it). |
| The SMOG Index | $1.043\sqrt{no.ofpolysyllables{\times\frac{30}{total sentences}+3.1291}}$ | Represents the grade level needed to read the text (e.g. 9.3 = a ninth grader can read it). |
| Automated Readability Index | $4.71(\frac{total characters}{total words})+0.5(\frac{total words}{total sentences})-21.43$ | Represents the grade level needed to read the text (e.g. 9.3 = a ninth grader can read it). |
| The Coleman-Liau Index | $0.0588L-0.296S-15.8$  $L=mean number of characters per 100 words$  $S=mean number of sentences per 100 words$ | Represents the grade level needed to read the text (e.g. 9.3 = a ninth grader can read it). |
| Linsear Write Formula | For each "easy word", defined as words with 2 syllables or less, add 1 point.  For each "hard word", defined as words with 3 syllables or more, add 3 points.  Divide the points by the number of sentences in the 100-word sample.  Adjust the provisional result $r$:   - If $r>20,Lw=r/2$ - If $r\leq20,Lw=r/2-1$ | Represents the grade level needed to read the text (e.g. 9.3 = a ninth grader can read it). |
| Dale-Chall Readability Score | $0.1579(\frac{difficult words}{total words}\times100)+0.0498(\frac{total words}{total sentences})$ | Represents the grade level needed to read the text (e.g. 9.3 = a ninth grader can read it). |
| Readability Consensus | Based on all the tests, returns the estimated school grade level required to understand the text. | Represents the grade level needed to read the text (e.g. 9.3 = a ninth grader can read it). |
