# Supplementary material for: Analyzing Suicide Risk From Linguistic Features in Social Media: Evaluation Study
Source: JMIR Form Res. 2022 Aug 30;6(8):e35563. doi: 10.2196/35563 (PMC9472054; doi:10.2196/35563)
Supplement: Multimedia Appendix 11 [file formative_v6i8e35563_app11.docx]

|  | Precision | Recall | F1-Score | Support |
| --- | --- | --- | --- | --- |
| Gradient Boost | 0.89 | 0.59 | 0.71 | 125 |
| Random Forest | 0.86 | 0.81 | 0.83 |  |
| Support Vector Machine | 0.97 | 0.41 | 0.58 |  |
