# Supplementary material for: Analyzing Suicide Risk From Linguistic Features in Social Media: Evaluation Study
Source: JMIR Form Res. 2022 Aug 30;6(8):e35563. doi: 10.2196/35563 (PMC9472054; doi:10.2196/35563)
Supplement: Multimedia Appendix 12 [file formative_v6i8e35563_app12.docx]

|  | Precision | Recall | F1-Score | Support |
| --- | --- | --- | --- | --- |
| Gradient Boost | 0.34 | 0.74 | 0.46 | 53 |
| Random Forest | 0.43 | 0.51 | 0.47 |  |
| Support Vector Machine | 0.31 | 0.95 | 0.47 |  |
